# Primary care in an unstable security, humanitarian, economic and political context: the Kurdistan Region of Iraq

**DOI:** 10.1186/s12913-017-2501-z

**Published:** 2017-08-23

**Authors:** Ali R. Shukor, Niek S. Klazinga, Dionne S. Kringos

**Affiliations:** Academic Medical Center, University of Amsterdam, Meibergdreef 9, 1105 AZ Amsterdam, The Netherlands

**Keywords:** Kurdistan region, Iraq, Primary care, Health system, Development

## Abstract

**Background:**

This study presents a descriptive synthesis of Kurdistan Region of Iraq’s (KRI) primary care system, which is undergoing comprehensive primary care reforms within the context of a cross-cutting structural economic adjustment program and protracted security, humanitarian, economic and political crises.

**Methods:**

The descriptive analysis used a framework operationalizing Starfield’s classic primary care model for health services research. A scoping review was performed using relevant sources, and expert consultations were conducted for completing and validating data.

**Results:**

The descriptive analysis presents a complex narrative of a primary care system undergoing classical developmental processes of transitioning middle-income countries. The system is simultaneously under tremendous pressure to adapt to the continuously changing, complex and resource-intensive needs of sub-populations exhibiting varying morbidity patterns, within the context of protracted security, humanitarian, economic, and political crises. Despite exhibiting significant resilience in the face of the ongoing crises, the continued influx of IDPs and Syrian refugees, coupled with extremely limited resources and weak governance at policy, organizational and clinical levels threaten the sustainability of KRI’s public primary care system. Diverse trajectories to the strengthening and development of primary care are underway by local and international actors, notably the World Bank, RAND Corporation, UN organizations and USAID, focusing on varying imperatives related to the protracted humanitarian and economic crises.

**Conclusions:**

The convergence, interaction and outcomes of the diverse initiatives and policy approaches in relation to the development of KRI’s primary care system are complex and highly uncertain. A common vision of primary care is required to align resources, initiatives and policies, and to enable synergy between all local and international actors involved in the developmental and humanitarian response. Further research that integrates the knowledge synthesized in this article, and enables actors in KRI to learn from their own experiences and efforts, along with those of other jurisdictions, would be invaluable towards the ongoing development of primary care.

## Background

Kurdistan Region of Iraq (KRI) presents a particularly idiosyncratic case for the analysis of primary care system development within the ongoing context of multiple interacting crises.

Culturally, KRI is part of *‘Greater Kurdistan’* (spanning southeastern Turkey, northern Syria and northwestern Iran), the homeland of Kurds, one of the largest stateless groups of indigenous peoples in the world. KRI is also comprised of sizeable and relatively peacefully-coexisting ethnic and religious minorities, notably Arabs, Yazidis, Shabaks, Assyrians, Armenians, Turkmen, Kakais and Mandaeans. Following eight decades of intermittent conflict with the Iraqi state, culminating in the Anfal genocide of the 1980s and imposition of the 1991 ‘No Fly Zone’, Kurds in Iraq gained de facto *autonomy. This transformed into de jure* semi-autonomous federal status after the US invasion of Iraq in 2003 [1, 2].

This semi-autonomous status was coupled with good security and significant petroleum-based economic rent, generating large revenues for the governing political parties. Exhibiting various characteristics associated with a “*resource curse*”, KRI was virtually transformed into an economic dependency of neighboring Turkey and Iran, and trickle-down economic growth (via salaries of a large public sector) was coupled with less democracy, increased corruption and poor institutional development [3].

Economic growth came to a grinding halt in 2014, with the simultaneous confluence of four interconnected crises: a *security crisis* stemming from the war with the Islamic State (IS); a *protracted humanitarian crisis* with the influx of close to two million Iraqi Internally Displaced Persons (IDPs) and Syrian refugees; an *economic crisis* stemming from a political stalemate with the Iraqi national government, leading to a freeze on oil production transfer payments (exacerbated by a drop in oil prices); and an *internal political crisis* between political parties in Kurdistan Region over power and resources, resulting in the increasing alienation and discontent of the citizenry [3, 4–6].

The continuous influx of refugees and IDPs, the vast majority of whom reside in community settings, have increased KRI’s population by nearly a third, and have significantly altered the region’s demographic and ethnic composition (see Table 1) [5]. This situation, coupled with the other crises, has resulted in severe pressures on KRI’s primary care system’s ability to meet the resource-intensive needs of continuously changing diverse sub-populations exhibiting extremely dynamic and complex epidemiological profiles [4, 7]. Table 1 provides an overview of a selection of demographic, economic and health care indicators of KRI.

**Table 1:** Selection of demographic, economic and health care indicators (KRI)

Population	5,472,436 (2015) [6]
Iraqi IDPs	958,344 (Aug 2016, *figure not including IDPs in disputed territories*) [54]
Syrian refugees	234,228 (Aug 2016) [54]
Military casualties	> 1500 Peshmerga and Kurdish security forces (since 2014) [92]
Annual budget transfer from Baghdad	17% of oil revenues (~ $12 billion/year) [7]
Actual budget received from Baghdad	~ $2 billion (total since 2014) [7]
Funds required to offset the impact of IDPs on KRI residents	$1.48 billion / year (2015) [7]
KRI Debt	$17 billion [20]
Budget deficit	$3.2 (2015); $2 billion (projected, 2016) [20]
Austerity program fiscal consolidation	37% of GRP (between 2014 and 2016) [20]
Poverty rate	3.5% (2012); 12% (2015, KRSO) [7]
Corruption index	High (KRI received a score of mid-30s, where 0 is considered corrupt and 100 clean. Comparison: Iraq received a score of 10). (EIU 2014) [3]
Theoretical annual public health sector budget (projected from pre-crisis levels)	$995.4 million (2015) [7]
Actual public health sector budget	$179.9 million (2014) [7]
Theoretical per-capita public health expenditure (projected from pre-crisis levels)	$159.91 (4)
Actual per-capita public health expenditure	Total per-capita public health expenditure: $33.74 (2014); Primary health care per-capita expenditure: $5.80 [7]
Physicians	13 per 10,000 (2014) [6]
Neonatal mortality	9 per 1000 live births (2009); Comparison: Iraq 25 per 1000; WHO Eastern Mediterranean Region 35 per 1000 (2009) [22]
Infant mortality	28 per 1000 live births (2011); Comparison: Iraq 36 per 1000; WHO Eastern Mediterranean Region 57 per 1000 (2011) [22]
Child (under 5 yrs) mortality	32 per 1000 live births (2011); Comparison: Iraq 45 per 1000; WHO Eastern Mediterranean Region 78 per 1000 (2011) [22]
Immunization coverage, children 12–23 months (Measles and DPT3 respectively)	90% and 81% (2008); Comparison: Iraq 69% and 62%; WHO Eastern Mediterranean Region: 83% and 82% (2008) [22]
Underweight, wasting and stunting (children under 5 yrs)	7%, 5%, and 15%, respectively (2011); Comparison: Iraq 27.5% stunted (2011) [22]
Cholera outbreaks (years)	2007, 2008, 2012 and 2015 (contained)
Diabetes (Type 2)	6.2% (Sulaimani Governorate, 2011) [28]
Cancer	38/100,000 (2006); 61.7 /100,000 (2014) (Sulaimani Governorate) [29]

Against this contextual backdrop, prominent *foreign actors* have emerged and gained prominence in relation to their influence on KRI’s social, economic and political development – particularly, its primary care system. For example, the humanitarian crisis has led to the introduction of actors involved in a *development-oriented humanitarian emergency response*, namely the World Health Organization (WHO)-led ‘Humanitarian Response Plan’ (HRP) for IDPs, and the UN Refugee Agency (UNHCR) led ‘Regional, Refugee and Resilience Plan’ (3RP) for Syrian refugees [8, 9]. The economic crisis, effectively bankrupting KRI, has led to the introduction of an austerity and neoliberal *structural adjustment reform program* by the World Bank, building on consultancy work of RAND Corporation, with an emphasis on the development of primary care [10]. Simultaneously, other large scale health sector reform-related policies and programs focus on the *sustainable development* by targeting Universal Health Coverage (UHC) and Millennium Development Goals (MDGs) [11]. The convergence, interaction and outcomes of these policy approaches in relation to the development of KRI’s primary care system are complex and highly uncertain.

The purpose of this review is to provide a comprehensive description synthesizing the current state of primary care in KRI to help inform and align ongoing policy developments. Such a systematic analysis, to our knowledge, does not exist to date.

## Methods

The descriptive analysis of KRI’s primary care system was performed using a framework operationalizing Barbara Starfield’s classic model for health services research, highlighting the following key primary care system domains: policy context, health system structure and governance, financing and funding, health data and information systems, human resources for health, health technologies, organization and management, and performance attributes (of service delivery and receipt) [12, 13]. These domains are commonly used internationally in recent primary care frameworks [14–16].

A scoping literature review was performed by adapting the York approach outlined by Arksey and O’Malley [17]. The search strategy involved several literature sources, including peer-reviewed scientific journals, official reports and secondary documents. PubMed was searched using the terms “Iraq” and “Kurdistan” for scientific articles published between 1990 and the present (as 1991 marks the first year of KRI’s autonomy). The search terms were purposefully kept very broad, as the goal was to conduct a sensitive rather than a specific search.

A “grey” news and policy document search (using the keywords “Kurdistan” coupled with “healthcare”, “health care”, “primary care”, “primary health care” along with the framework’s domain keywords) was performed in English and Arabic using Google, along with searches of organizational websites, namely: governmental (e.g. Iraqi and KRG ministerial), international financiers (eg. World Bank), development agencies / NGOs (e.g. WHO Health Cluster, HRP, 3RP, ReliefWeb), bilateral organizations (e.g. USAID’s Development Experience Clearinghouse), academic organizations / think tanks (eg. RAND Corporation, Middle East Research Institute), international / local news agencies (e.g. Rudaw), and statistical databases (eg. Global Health Data Exchange). Where possible, website searches were performed in a systematic manner. Site maps, publication links and internal website search engines were leveraged, when available.

Snowball searches were performed by leveraging the references in key scientific articles and reports (ie. RAND Corporation reports).

The first author, a Canadian PhD student of Kurdish ethnicity, fluent in both English and Arabic, performed the initial selection and review of relevant literature. The first author’s Kurdish language skills were sufficient to identify and interpret articles of potential relevance, which were forwarded to fluent Kurdish-speaking colleagues for validation of interpretation. Inclusion and exclusion criteria related to whether literature contained meaningful content in relation to operationalizing the framework. Included literature was categorized and catalogued in folders, respectively titled by each of the framework’s domains.

Themes and data from the literature were organized and synthesized according to the key primary care system domains using thematic content analysis [12]. Findings were compiled and triangulated against various data sources.

Synthesized findings were shared with the two co-authors, who are experienced health system researchers. The co-authors contributed to the analysis, and provided guidance regarding the initiation of additional searches to enable the completeness and validation of findings and conclusions.

To further complete and validate the data, consultations were conducted with three experts on primary care in KRI, all fluent in Kurdish. The experts included an Assistant Professor of Public Health at a College of Medicine in KRI, a KRI think tank Research Fellow, and a WHO Public Health Officer.

## Results

The following primary care domains are described: policy context, health system structure and governance, financing, funding, health data and information systems, human resources for health, health technologies, organization and management, performance attributes of primary care delivery and receipt (ie. access, continuity of care, comprehensiveness and coordination of care), health outcomes, and patient and provider experiences.

### Policy context

#### Summary points

- *➢ RAND Corporation has been commissioned by the KRG’s Ministry of Planning since 2010 to produce analyses of KRI’s health care system (with an emphasis on primary care), and provide evidence-based policy recommendations.*
- *➢ The World Bank has developed a structural economic reform program for KRI, drawing from the work of RAND Corporation.*
- *➢ UN’s Sustainable Development Goals and Universal Health Coverage (UHC) are being operationalized through programs such as the Iraq Public Sector Modernization (I-PSM) Program’s ‘Integrated District Health System Based on a Family Practice Approach’ (IDHS-FPA).*
- *➢ Millennium Development Goal (MDG) targets has been the focus of bilateral agencies such as the US Agency for International Development (USAID) in KRI, concentrating on primary care and reducing maternal and child mortality.*
- *➢ UN agencies and their partners are operationalizing the Humanitarian Response Plan (HRP) and Regional, Refugee and Resilience Plan (3RP) programs, which are being oriented around sustainable development.*

A recent survey of KRI’s health policy makers and advisors indicated that international conferences, seminars and foreign consultants were the main sources of evidence used for policy making in the region [18]. Indeed, the employment of high profile international consulting and lobbying organizations has been central to the development of primary care policy in KRI since 2008, when the Kurdistan Regional Government (KRG) requested assistance with the development of a ‘Regional Health Master Plan’ from Dr. Bernard Kouchner, co-founder of Doctors Without Borders (MSF), and French Minister of Foreign Affairs at the time [19]. It should be noted that Dr. Kouchner was instrumental in lobbying for the creation of a no-fly zone in Northern Iraq in 1991, that helped form what is now the semi-autonomous KRI.

From this lens, the commissions can be viewed as purposive actions from the KRG to establish independence in policy development from the Iraqi central government during times of rapid social and economic development. RAND Corporation has been particularly influential, commissioned by the KRG’s Ministry of Planning since 2010 to produce analyses of KRI’s health care system (with an emphasis on primary care), and provide evidence-based policy recommendations. The economic and humanitarian crises have further increased the KRG’s reliance on foreign actors, particularly the World Bank, RAND Corporation, the WHO and USAID – all of whom focus efforts in relation to the development of primary care, albeit with differing strategies and goals [7, 20].

#### Structural adjustment

The confluence of the political, economic, security and humanitarian crises have resulted in severe socioeconomic pressures on KRI [20]. Effectively bankrupt, the KRG has implemented a cross-cutting public sector austerity program (a fiscal consolidation equivalent to 37% of GDP since 2014) [20]. In 2016, the KRG requested the World Bank to develop and implement a structural economic reform roadmap [10].

The neoliberal reform program’s cross-cutting health sector policy priority areas and actions relate to: 1) *introducing a sound health care financing system*; 2) *improving the availability and quality of clinical services*; 3) *promoting preventive services*; and 4) *improving public sector management, regulatory and policy capacity* [10, 21]. The priorities draw from work by RAND Corporation, which has been actively commissioned since 2010 by the KRG to lead health services research and policy-related activities, with an emphasis on the development of a primary care system [18, 22–24]. RAND is officially listed as the World Bank’s key “*potential international partner and technical assistance*” in relation to health sector reform under the structural adjustment program [10].

It should be noted that a ‘semi-private’ system was implemented at the hospital level as of 2012, but may also be applied to primary care. The initial basic premise of the ‘semi-private’ system was that patients should pay 20% of the market cost of public care. After 2014 the share of patient was reported to have increased to 80%, and in some cases to 100% [25].

#### Enabling universal health coverage (UHC)

Achieving the UN’s Sustainable Development Goals have been the focus of authorities and international development organizations in Iraq and KRI [11, 26]. A notable example is the *Iraq Public Sector Modernization (I-PSM) Program* – a $55 million four-year program funded by the EU and supported by eight UN agencies [27]. The I-PSM’s health care policy roadmap is operationalized through the WHO’s flagship initiative: the ‘*Integrated District Health System Based on a Family Practice Approach*’ (IDHS-FPA), which aims to operationalize the WHO’s overall strategic plan relating to primary care reform and revival in low and middle income countries, towards achieving UHC [2, 8, 22]. Iraq’s Basic Health Services Package (BHSP) is frequently mentioned in such programs as the tool to achieve UHC. It was also one of the benchmarks recognized by the World Bank, IMF and the UN in the International Compact with Iraq in 2007 [33]. Pre-implementation evaluations (ie. facility surveys) of the IDHS-FPA have demonstrated significant interest and buy-in from regional policymakers and primary care providers [30, 31].

Iraq’s National Health Policy (NHP 2014–2023), developed in collaboration with the WHO, aims “to move the Iraqi health sector agenda towards UHC”, thereby setting a broad policy context that shifts from emergency responsiveness towards sustainable development [32]. However, despite referring to cooperation with the KRG in the NHP’s development, little evidence of meaningful strategic or content alignment exist.

#### Achieving millennium development goals (MDGs)

Leveraging and strengthening primary care, towards achievement of MDG targets, has been the focus of bilateral agencies such as the US Agency for International Development (USAID) in KRI. The $75 million four-year USAID Primary Health Care Project in Iraq (PHCPI) focused on assisting the Iraqi and KRG Ministries of Health (MOHs) in relation to improving primary care and reducing maternal and child mortality, towards achieving Millennium Development Goals (MDGs) related to child mortality and maternal health [33]. The PHCPI focused on three key components: 1) strengthening primary care health management systems, 2) improving the quality of clinical services (primary care performance), and 3) encouraging community involvement to increase the demand for and utilization of primary care [34]. The PHCPI was concluded at a joint USAID-Iraqi MOH conference in 2015. It is unclear whether a final comprehensive evaluation of the program was ever conducted [35].

#### Development-oriented approaches to humanitarian crises

National authorities, international donors, aid agencies and NGOs are increasingly framing protracted humanitarian crisis activities within developmental frameworks, with a focus on building resilience within refugee, IDP and host communities and settings [36]. UN agencies and their partners are therefore operationalizing policies associated with the Humanitarian Response Plan (HRP) and Regional, Refugee and Resilience Plan (3RP) programs orienting humanitarian activities as opportunities and enablers of sustainable development [37, 38]. Both programs incorporate targeted resilience interventions aimed at the self-reliance of refugees, IDPs and host communities and institutional capacity building (30% of the 3RP’s budget is dedicated to resilience) [9]. The content of the HRP and 3RP’s development-oriented approaches increasingly incorporate market-led development praxis, incorporating and framing the private sector as development actors, while transforming the traditional roles of international development and humanitarian agencies [36, 39].

### Health system structure and primary care governance

#### Summary points

- *➢ The autonomous KRG MoH oversees six Directorates of Health, which in turn are comprised of Districts and Sub-Districts.*
- *➢ Little is published in relation to primary care governance structures, capacities and processes in either KRI or Iraq, or the nature of interactions between them.*
- *➢ The humanitarian crisis has led to an influx of a large number of international NGOs and donors, whose activities are mainly coordinated under the frameworks of the HRP and 3RP.*
- *➢ The KRG’s Joint Crisis Coordination Centre (JCCC) aims to strengthen institutional governance, data monitoring and coordination capacity in relation to the humanitarian crisis response.*
- *➢ The private health market is poorly governed and regulated. The structural adjustment program aims to enhance enhanced public oversight and regulation of KRI’s rapidly developing private health care sector.*

#### Public health sector structure and primary care governance

The autonomous KRG MoH, established in the early 1990s, generally follows the basic centralized bureaucratic organizational structure of the Iraqi MOH [2, 19, 22]. The KRG MOH oversees six Directorates of Health (DoH Slemany, Halabja, Erbil, Duhok, Garmian and Raparin) - each led by a Director General), which in turn are comprised of Districts and Sub-Districts. Organizational charts exist at ministry, governorate and district levels; however, detailed descriptions of their content in relation to key roles, responsibilities and functions in health care including primary care are lacking. Little is published in relation to primary care governance structures, capacities and processes in either KRI or Iraq, or the nature of interactions between them [19, 22, 24].

Leadership positions (ie. health directors at regional, district or facility levels) in health care, including primary care, have often been comprised of specialist clinicians with limited leadership, management or administrative training [40, 41]. There has been a recent trend for younger, less well-established specialist clinicians to assume leadership positions as Director Generals of Health [25]., This complicates the development of governance structures supportive to the primary care system. However, in recent years, hospital-centric health care policy rhetoric has shifted towards an emphasis on primary care, and “*support from the Directorates of Health and presence of real intentions to improve primary health care services*” has been reported [21, 41].

Management systems seem to be ad hoc in nature and vary greatly across governorates, districts, and facilities [22]. Employment is often characterized by non-standardized selection processes, which is particularly problematic, as public primary care physicians are salaried civil servants [3]. Recruitment to the civil service has been reported to be rarely done through standardized merit-based selection processes. Most KRG civil servants (54%) have received no training at all for their role, including on corruption risks [3].

This weak governance situation is intriguing in light of targeted large-scale international projects specifically aimed at reforming and strengthening governance capacities, structures and processes across the entire health sector. These include the I-PSM program, the UNDP-led ‘*Local Area Development Program’*, and three USAID programs. As previously described, a core component of the World Bank’s structural adjustment program relates to health sector management reform, to be operationalized in partnership with RAND Corporation [27, 42, 43].

Further challenging health system governance is the humanitarian crisis, which has led to an influx of a large number of international NGOs and donors, whose activities are mainly coordinated under the frameworks of the HRP and 3RP. The HRP is comprised of 15 UN agencies, funds and programs working in partnership with over 170 international and national partners [38]. The 3RP is comprised of a large partnership of international and local governmental agencies and NGOs [44]. Both programs operationalize coordination and accountability mechanisms to align and rationalize governance decisions and activities [38, 44]. In 2015, the KRG Ministry of Interior inaugurated the *Joint Crisis Coordination Centre* (JCCC), with the aim of strengthening institutional governance, data monitoring and coordination capacity in relation to the humanitarian crisis response [45].

#### Private health sector governance

The KRG’s MoH periodically issues rules and regulations with the aim of regulating the private health market, which are both weak and inadequately implemented. The main reason has been reported to be the existence of conflicts of interest among physicians and managers who are responsible for developing and enforcing such systems, who simultaneously have shares or own enterprises in the private health care market [25].

A core component of the structural adjustment program relates to enabling enhanced public oversight and regulation of KRI’s rapidly developing private health care sector [10]. The program builds on the momentum of the KRG’s 2006 Investment Law and work of the Kurdistan Board of Investment, which have actively promoted public-private partnerships.

The emphasis on oversight and regulation stem from RAND’s observation of the “*unguided growth of private sector health care*… *rapidly expanding without regulatory guidance or a strategic investment process*” [46]. It has been reported that “*the lack of top-down regulatory bodies leaves a health system that is de facto governed by physicians*” [47].

### Financing primary care

#### Summary points

- *➢ The KRG is legally entitled to receive a proportion of Iraq’s overall public budget (a population-based resource allocation, set at 17%).*
- *➢ KRG public spending on health as a proportion of total government expenditure has been reported to be between 4.8–5.5%, similar to that of Iraq and regional countries. An estimated 20% of the public health care budget is allocated to primary care.*
- *➢ The WHO National Health Accounts (NHA) has not generated sufficiently accurate or updated figures for KRI, or Iraq in general.*
- *➢ Due to the ongoing crises, the KRG is running high annual deficits, with an estimated total debt of $17 billion as of January 2016. When factoring in the influx of refugees and IDPs, per capita health expenditure has been estimated to have decreased from $159.91 (pre-crisis) to $29 (2014). Actual per capita spend for public primary care decreased from $24.89 (2012) to $5.80 (2014).*
- *➢ The overwhelming majority of the budget is dedicated to salaries. Facility budgets and staff salaries are not linked to resource utilization, activity or performance.*
- *➢ Private health care facilities are financed largely by direct out-of-pocket payments (estimated at 39.7% of total health spend in 2014). These figures, however, are not accurate or appropriately disaggregated.*
- *➢ As of January 2016, funding the humanitarian response has been extremely limited (eg. only 35% of the 3RP’s health sector component has been funded).*
- *➢ A core component of the World Bank’s structural adjustment program relates to health system financing and funding reform. RAND has outlined a strategic vision and road map, moving initially towards a tax-based national health service, followed by the development of a social health insurance system.*

#### Public sector financing

The KRG is legally entitled to receive a proportion of Iraq’s overall public budget (a population-based resource allocation, set at 17%), which is overwhelmingly based on oil sale revenues [4]. The KRG Ministry of Finance and Ministry of Planning determine the overall public health budget [22]. KRG public spending on health as a proportion of total government expenditure has been reported to be between 4.8–5.5%, similar to that of Iraq and regional countries [7, 22, 48]. The World Bank estimates that approximately 20% of the public health care budget is allocated to primary care (~$126 million in 2012) [7].

Patient co-payments and user fees have historically been minor, and have not represented a significant source of revenue for the public health care system [40, 41]. However, more recently a ‘semi-private’ scheme has been reported to have been applied to hospitals, which increased out-of-pocket expenditure in public hospitals to approximately 80%. The current economic crisis with the reduced income of the general population has shifted many patients to seek care in public facilities rather than private ones. The load on public facilities has increased as a result [25].

#### National Health Accounts (NHA)

Since 2009, in support of the I-PSM program, the WHO has worked with Iraqi authorities to implement the National Health Account (NHA) tool [48]. The NHA is used by over 100 low and middle income countries to inform evidence-based decision-making in relation to system financing and resource allocation, and enable cross-country comparisons. The NHA, however, has not been able to generate sufficiently accurate or updated figures for KRI, or Iraq in general. A footnote of the latest NHA report highlights that “*estimates should be viewed with caution, as they are derived from scarce data*”, and “*do not include expenditures for Northern Iraq*” [48]. Whereas the 2008 Iraq NHA was based on data from validated surveys, the latest NHA data is generally derived from estimates by the WHO or Iraq Permanent Mission in Geneva, of unknown methodology [49].

#### Economic crisis

Due to the ongoing crises, the KRG is running high annual deficits ($3.2 billion in 2015), with an estimated total debt of $17 billion as of January 2016 [20]. The KRG MOH has received only 21.1% of their annual expected budget allocation in 2014 ($179.9 of $852.7 million) [7]. When factoring in the influx of refugees and IDPs, per capita health expenditure has been estimated to have decreased from $159.91 (pre-crisis) to $29 (2014). Actual per capita spend for public primary care decreased from $24.89 (2012) to $5.80 (2014). The total stabilization cost required to bring per capita health expenditure back to baseline (2011) levels was estimated extra $317.1 million (2015) [7].

#### Public sector funding

Public health care facilities are funded through traditional line-item budgeting processes. The KRG’s Directorates provide the MOH with budgetary needs assessments that are jointly reviewed and translated into operational budgets. Funds approved by the KRG MOH generally flow directly from the KRG Ministry of Finance to the Governorates of Health [19, 22].

The overwhelming majority of the budget is dedicated to salaries [22, 24]. Facility budgets and staff salaries are not linked to resource utilization, activity or performance. The public payroll has been reported to include ghost employees, with salary structures bloated by scores of allowances, along with lifetime pension schemes [24].

The KRG also pays for care to be received abroad, in cases where services are locally unavailable. It is unclear whether standardized criteria are used to underpin such decisions. The majority of those who seek medical treatment abroad are funded by specially established committees that are directly linked to the offices of the ruling political parties. According to experts, decisions to offer funding are mainly guided by political consideration rather than medical need [25].

#### Private sector financing

Private health care facilities are financed largely by direct out-of-pocket payments [48]. The latest Iraq NHA estimates private expenditure as a percent of total health spend to be 39.7% in 2014, entirely comprised of out-of-pocket expenses (the 2014 Iraqi National Health Policy quotes the figure at 41%) [32, 48]. These figures are not accurate or appropriately disaggregated. A few private health insurance schemes have recently been offered in KRI, which mainly cover expats and local employees of international organizations and companies [25].

Accurate disaggregated statistics related to catastrophic health expenditures do not exist.

#### International NGO and donor funds for primary care

Particularly important sources of revenue for primary care services, particularly in relation to refugees, IDPs and host communities are derived from initiatives related to the HRP and 3RP. However, as of January 2016, funding the humanitarian response has been limited (ie the HRP and 3RP have only been funded at 56% and 30%, respectively). Only 35% of the 3RP’s health sector component has been funded [8, 9, 44].

#### Financing reform

A core component of the World Bank’s structural adjustment program relates to health system financing and funding reform, to be operationalized in partnership with RAND Corporation. RAND has outlined a strategic vision and road map involving a comprehensive two-phase financing reform program, moving initially towards a tax-based national health service, followed by the development of a social health insurance system [10, 24].

### Health data and information systems

#### Summary points

- *➢ There is a serious paucity of timely and accurate health-related statistics in KRI. Three main sources of data exist: surveys; a KRG Health Management Information System (HMIS); and data collected through humanitarian programs such as the 3RP and HRP.*
- *➢ Surveys conducted by national and international agencies are generally outdated and poorly disaggregated, and are therefore of limited present value.*
- *➢ RAND recommended interventions to develop and improve health information systems in KRI.*
- *➢ Both the HRP and 3RP programs conduct regular assessments in relation to the health needs of refugees, IDPs and host communities, along with health care supply and utilization.*

The economic and humanitarian crises have resulted in rapidly changing epidemiological and population profiles in KRI, with poverty rates more than doubling in recent years [4, 20]. In light of this catastrophic situation, the paucity of timely and accurate health-related statistics is particularly striking. Three main sources of data exist: surveys; a KRG Health Management Information System (HMIS); and data collected through humanitarian programs such as the 3RP and HRP [22, 50].

#### Surveys

Surveys conducted by national and international agencies are generally outdated and poorly disaggregated, and are therefore of limited present value, due to recent rapid socioeconomic and epidemiological changes. The *Global Health Data Exchange (GHDx)* highlights some of the main survey instruments that are regularly used and cited [50]. The latest relatively reliable survey are UNICEF’s Multiple Indicator Cluster Surveys (MICS 4) from 2011. MICS 5 was originally planned for 2016, and is now planned for 2017, but will probably not be conducted, according to experts [25].

#### Health management information system (HMIS)

A HMIS is used to collect and send clinical registry data from the Directorates to the MOH [22, 24, 46]. This data is usually presented in the MoH Baghdad annual reports and DoH annual reviews. However, significant limitations related to both the content and quality of statistics hinder its utility. Patient record-keeping (mainly paper-based) is mostly non-existent. The Ministries of Planning (Kurdistan Regional Statistics Office) and MOH both contain central data collection and analysis units; however, staff have been reported to be inadequately trained to enter, process and analyze data [22, 40, 41]. Health care needs are often indirectly perceived by expressed demand, which is exacerbated by physician-induced demand [19].

An ongoing cooperation between the KRG, the Italian Ministry of Foreign Affairs and the University of Tor Vergata aims to set up a system of health monitoring and surveillance [51]. The system aims to collect health data routinely, which would be accessible from all PCs and Mobile Devices in KRI. The project also includes training activities, addressed to local healthcare professionals and statisticians, focused on the implementation of the Health Information Systems, on the International Classification of Diseases (ICD) and on epidemiological surveillance. It is, however, unclear whether the KRG has the resources and capacity to run and maintain the program [25].

RAND recommended interventions to develop and improve health information systems in Kurdistan Region, namely 1) the development and implementation of management information systems (MIS) to monitor clinic resources, services and utilization, and 2) the development of enhanced public health surveillance and response systems [52, 46, 53].

#### Humanitarian agency data collection

Both the HRP and 3RP programs conduct regular assessments in relation to the health needs of refugees, IDPs and host communities, along with health care supply and utilization. These include multi-sector and multi-agency needs assessments, an *ActivityInfo Dashboard* inter-agency planning, monitoring and evaluation tool, and regular reports by the UN Office for the Coordination of Humanitarian Affairs (OCHA), the WHO Health Cluster and data from the International Organization for Migration (IOM) Displacement Tracking Matrix (DTM) [54, 38, 44].

Furthermore, there are local individual and collaborative efforts, such as Hawler Medical University’s collaboration with the London School of Economics (LSE), which aims to estimate death rates and the health status of IDPs in camps, using a retrospective household survey [55].

### Human resources for health

#### Summary points

- *➢ Accurate, disaggregated numbers relating to the total number and distribution of primary care physicians (by professional type, organizational setting, geography) are generally unavailable. The proportion of primary care physicians to specialists across KRI is unknown.*
- *➢ Primary care physicians are not equitably distributed in accordance to population health needs, with severe shortages in rural areas.*
- *➢ Physicians staffing public primary care centers are primarily recent medical graduates, with limited formal training.*
- *➢ Since August 2014 with the ISIS threat and the followed economic crises, many young physicians have left KRI seeking jobs in Europe, resulting in physician shortages.*
- *➢ Medical education is generally based on the standard Iraqi curriculum based on the six year traditional British curriculum. However, schools within KRI are increasingly integrating small group teaching and problem-based learning.*
- *➢ Since 2006, four-year family and community medicine specialties have been recognized and developed by the Kurdistan Board of Medical Specialties (KBMS).*
- *➢ The KRG’s Ministry of Higher Education has invested heavily in continuous medical education, training and professional development activities through its $100 million USD ‘Human Capacity Development Program’ (HCDP).*
- *➢ Accurate statistics of the number and distribution of nurses working in KRI’s primary care centers are unavailable. Key problems exist in relation to distribution, qualifications, competencies and experiences.*

### Primary care physicians

#### Numbers, types and distribution

The overall number of physicians per capita in KRI (13 per 10,000 in 2014) has been reported to be nearly double that of the rest of Iraq, and significantly less than neighboring countries [6]. Accurate, disaggregated numbers relating to the total number and distribution of primary care physicians (by professional type, organizational setting, geography) are generally unavailable. The proportion of primary care physicians to specialists across KRI is unknown.

Evidence points to the fact that most physicians are specialized, working in private practices in urban settings. For example, the number of physicians per capita working in Erbil governorate’s public primary care and hospital facilities was reported to be 5.1 per 10,000 [56]. 83.6% of those physicians were deployed in urban areas, and only 23.3% worked in Primary Health Care Centers (PHCCs) (74.2% worked in hospitals) [56].

Primary care physicians are not equitably distributed in accordance to population health needs. Significant variation has been reported in relation to physician distribution between and within Governorates, with severe shortages in rural areas [19, 40, 53]. Physicians servicing rural and remote regions have been reported to be inexperienced compared to urban peers, and are not properly supervised or mentored [53].

Physicians staffing PHCCs are primarily recent medical graduates, with limited formal training, apart from post-graduate residencies and 1 year of obligatory work in rural PHC sub-centers [40, 41]. A small number of community and family medicine specialists exist, but are reported to be unwilling to work in PHCCs [25]. A high proportion of specialists have been reported to be working in urban PHC centers, as local hospitals are unable to accommodate a growing number of specialists [40, 41].

Perhaps the only scientific study of the types and distribution of physicians in KRI was performed in Erbil Governorate in 2010 [56]. PHCCs there were staffed by “rural practitioners” (35.3%), general practitioners (24.1%), “specialty practitioners” (33.5%) and specialists (7.1%, including consultants). Updated studies that further define the types and distribution of professional categories within PHCCs across KRI are required [56].

It should be noted that specialized physicians fleeing Iraq currently work in KRI; however, accurate figures relating to their numbers, distribution, functions and work settings are unavailable [40, 41, 57, 58]. Since August 2014 with the ISIS threat and the followed economic crises, many young physicians have left KRI seeking jobs in Europe, primarily in Germany. This has been attributed to physician shortages, particularly in Duhok [25].

#### Education and training

Medical education is generally based on the standard Iraqi curriculum based on the 6 year traditional British curriculum [59]. However, schools within KRI are increasingly integrating small group teaching and problem-based learning [59, 60]. Several medical schools are recognized by the UK’s General Medical Council; furthermore, UK academic institutions regularly participate in conducting examinations of final year medical students.

Following graduation, students are required to complete 2 years of three-month postgraduate residency rotations within hospitals. Following this period, physicians must complete 1 year of obligatory service in (mainly rural) PHC Sub-Centers, with little supervision [53, 59].

#### Postgraduate activities and continuous medical education (CME)

KRI’s medical colleges provide higher diploma study opportunities in several clinical specialties and subspecialties, along with postgraduate MSc and PhD programs. Since 2006, four-year family and community medicine specialties have been recognized and developed by the Kurdistan Board of Medical Specialties (KBMS), offered by three training centers at KRI’s largest medical colleges [61]. There are efforts by the DoHs and MoH to get more physicians specialized in family medicine through opening more seats of postgraduate studies in family medicine (higher diploma and board); however, there are no reforms in the health system to accommodate them after graduation [25].

The KRG’s Ministry of Higher Education has also invested heavily in continuous medical education, training and professional development activities through its $100 million USD ‘*Human Capacity Development Program*’ (HCDP), which supports split-site PhDs, post-doctoral development, continuous medical education and collaborative research activities [62].

### Nursing

#### Numbers and distribution

Accurate statistics of the number and distribution of nurses working in KRI’s PHCCs are unavailable. Key problems, however, have been reported to be less related with the total number of nurses or gender distribution, but with their distribution, qualifications and competencies [53].

#### Education & training

Kurdistan Region’s nursing colleges offer four-year undergraduate nursing (BScN) degrees [63]. Curricula integrate the basic sciences, the humanities and social sciences, with clinical experience in specialized and community care settings. Nursing colleges have further developed their education programs through the creation of formal academic and scientific linkages with international universities and scientific events [63]. Technical institutes also offer nursing programs. There is also a new program for BSc midwifery; however, integrating the new graduates to the health system has been reported to be a main challenge [25].

#### Postgraduate and continuous medical education (CME)

Research in the field of nursing is being promoted, with new MSc and PhD programs being offered (eg. via the HCDP) [63, 64]. CME is not well developed, and is generally limited outside of academic settings, especially in rural and remote areas [64]. Several large-scale international educational and professional development programs have also operated in KRI (eg. by the PHCPI Nursing Task Force, WHO’s Nursing, Midwifery & Allied Health and International Council of Nurses). There are also some NGO efforts; for example, Latter Day Saints charity recently established the Erbil nursing and midwifery development program in collaboration with Hawler Medical University and DoH Erbil [25].

### Health technologies (drugs, devices and supplies)

#### Summary points

- *➢ Governance capacities and processes relating to the domain of health technologies are weak.*
- *➢ The weak governance situation has manifested troublesome phenomena relating to the local manufacturing, importation and distribution of expired and counterfeit pharmaceuticals, which has been associated with significant morbidity, mortality and anxiety among the population.*

#### Governance, regulation and quality control

Governance capacities and processes relating to the domain of health technologies are extremely weak [48–50]. The roles, responsibilities and relationships of the KRG MOH in Erbil, the *Kurdistan Medical Control Agency (KMCA)*, Kurdistan Syndicate of Pharmacists, Iraqi MOH (specifically its Directorate of Technical Affairs), Kimadia (The State Company for Marketing Drugs and Medical Appliances) and Iraqi Syndicate of Pharmacists, are diffuse and unclear [65, 66, 67, 68].

The weak governance situation has manifested troublesome phenomena relating to the local manufacturing, importation and distribution of expired and counterfeit pharmaceuticals, which has been associated with significant morbidity, mortality and anxiety among the population [69]. The KMCA attempted to strengthen its pharmacovigilance and regulatory capacity by contracting a UK-based quality control company, which was subsequently investigated for its failure to meet basic contractual requirements [68, 70]. Furthermore, there exists no systematic monitoring in relation to the appropriate and rational utilization of health technologies within public clinics, resulting in antimicrobial resistance [71–73].

The WHO has been reported to be assisting the Iraqi central government to establish a national drug regulatory authority [74]. Furthermore, the World Bank structural adjustment program incorporates reforms related to “*pharmaco-vigilance (surveillance and regulatory oversight), with increased quality criteria, stricter rules for prescription, storage and distribution, and fight against counterfeiting drugs*.” [10]. CRDF global is also conducting training, research and capacity building in the field of biosecurity and biosafety of laboratories in KRI [25].

### Primary care service delivery

#### Summary points

- *➢ De jure, all Iraqi citizens can access KRI’s public health care services. Primary care services are formally specified under the “Basic Health Services Package” (BHSP) and “Essential Drug List” (EDL). However, actual services are limited by resource and supply-chain management constraints, along with a lack of knowledge relating to the existence of the BHSP and EDL.*
- *➢ The BHSP specifies the content of four primary care models, outlining their required service components, essential medicines, equipment, staffing, and support and supervision requirements at district and national levels. The BHSP, however, has not been operationalized in a systematic, consistent or evidence-based manner across KRI.*

#### Eligibility and scope of services

De jure, all Iraqi citizens (including IDPs) can access KRI’s public health care services [53, 75]. Foreigners are not afforded the same legal protection under Iraq’s constitution; however, de facto, Syrian refugees are allowed to access public health care (including dental) services [44, 76].

Primary care services are formally specified under the “Basic Health Services Package” (BHSP) and “Essential Drug List” (EDL), defined by the WHO and Iraqi MOH [75]. However, actual services are limited by resource and supply-chain management constraints, along with a lack of knowledge relating to the existence of the BHSP and EDL.

#### Basic health services package (BHSP)

Public primary care in KRI is provided through Primary Health Care Centers (PHCCs), which are theoretically modelled and categorized according to criteria of the BHSP, which outlines the minimum set of essential health services that the Iraqi population need to have guaranteed access to (see Table 2).

**Table 2:** Components of the Basic Health Services Package (BHSP)^a^ [75]

Maternal and newborn health services	Antenatal, delivery, postnatal, family planning, newborn care
Child health and immunization services	Growth monitoring (<5 yrs), immunization (WHO EPI), Integrated Mgmt of Childhood Illness (IMCI, <5 yrs), Standard case mgmt. ARI <5 yrs., ear problems, fever, diarrhea (<5 yrs), diarrheal symptoms, measles, malnutrition and anemia, vitamin supplementation, case mgmt. For infants <2 months)
Communicable disease control	Respiratory infections, gastrointestinal infections, amoebiasis, hemorrhagic fever, STIs, tuberculosis control (DOTS plus), HIV / AIDS, typhoid, hepatitis, leishmaniasis (CL + VL), schistosomiasis, meningitis
Nutrition interventions	IEC (information, education, communication), nutrition promotion, malnutrition prevention / treatment
Immunization	IEC, campaigns, disease surveillance and reporting
Non-Communicable Disease Control	IEC, health promotion, cardiovascular (hypertension, heart, cerebrovascular), diabetes mellitus, arthritis, gastrointestinal (peptic ulcer, chronic ulcerative colitis, urinary tract infections, skin diseases, malignancies, breast cancer, cervical cancer, rheumatic fever, common eye diseases, conjunctivitis, cataract, glaucoma, corneal opacity, common ear diseases, hearing loss, other common ear infections
Mental health	Education and awareness, psychosis (identification and biopsychosocial management), anxiety, depression, epilepsy, substance abuse, support, referral
Emergency care	Case management (ie. respiratory/cardiac), diabetic emergencies, trauma, poisoning, bleeding, obstetrics, allergic reactions
Food safety, environmental and school health	IEC, food safety, environmental health (ie. medical waste mgmt., water chlorination examination, student screening, vaccination
Health education	Health education campaigns, materials, media, social mobilization for health programs
Laboratory services	Hematology, serology, biochemistry, bacteriology (direct microscopy, staining smears, culture, rapid bacteriological test), parasitology, cytology
Imaging	X-rays (chest, abdomen, skeletal), Ultrasound, ECG
Essential medicines	Anesthetics (GA, oxygen, local anesthetics), Analgesics, antipyretics, NSAIDs, Anti-allergics and anaphylaxis medicines, anticonvulsants/anti-epileptics, anti-infective medicines (anti-heminthics, antibacterials, antituberculosis), antifungals, antiprotozoals, antileishmaniasis, urinary antiseptics, blood medicines (anti-anaemia, coagulation), blood products and plasma subs, cardiovascular drugs, dermatological, diuretics, gastrointestinal, hormones / endocrine, contraceptives, opthalmological preparations, oxytocics / antioxytocics, phychotherapeutics, respiratory, vitamins/minerals, vaccines
Equipment	Imaging, laboratory, dental unit, EPI (immunization unit), labour and delivery unit and WMO clinic, procedure room, emergency/casualty room, observation room, consultation / exam room,

^a^The complete list and detailed breakdown by PHC facility type is available in “A Basic Health Services Package for Iraq, 2010” [75]

The BHSP specifies the content of four primary care models, outlining their required service components, essential medicines, equipment, staffing, and support and supervision requirements at district and national levels (see Table 3) [75]*.*

**Table 3:** Primary care models defined in Iraq’s Basic Health Services Package (BHSP)

PHC Main Center (Category A) Catchment area: 10,000–30,000	Comprehensive PHC center, staffed by doctors, nurses, midwives and laboratory and pharmacy technicians. PHC main centres provide a wide range of preventive and curative services related to maternal & child health care, immunization, communicable diseases, non-communicable diseases, mental health, emergency care, general dentistry, laboratory services, and essential medicines.
PHC Main Center (Category B) 10,000–30,000	Same as Category A above, with the addition of a training facility. Staffed by doctors, nurses,midwives and laboratory and pharmacy technicians.
PHC Main Center (Category C) 10,000–45,000	Same as Category A, with the addition of uncomplicated emergency and obstetric care. Staffed by doctors, nurses, midwives and laboratory and pharmacy technicians.
PHC Sub-Centers (Type D) 5000–10,000	Not staffed by physicians; only trained health workers (nurses or paramedics, and a vaccinator). Services offered include preventive and basic curative services, simple diagnostic procedures and maternal & child health services.
Community Health Houses (CHHs)	Staffed by community health workers offering simple public health functions, micronutrient supplementation, vaccination support and referrals.

Cetorelli et al. (2014) reported that KRI had approximately four times the number of PHCCs than the rest of Iraq, as a proportion of the population (19.6 vs 5.4 PHCCs per 100,000 population in 2012) [77]. Furthermore, the decade after the US invasion saw an average increase of 4.3 PHCCs per 100,000 population in Kurdistan versus an average increase of only 1.4 in the rest of Iraq [77]. A KRI think tank recently highlighted the importance of the operationalization and development of the BHSP for the development of the content of primary care in KRI [78]. The BHSP is indeed a central component of KRG and UN agency initiatives related to strengthening primary care, which have highlighted the need to review its content against contemporary evidence (eg. cost-effectiveness), KRI’s changing epidemiological profile, and resource limitations, and to translate its content to practical staff guidelines [30, 31].

The BHSP, however, has not been operationalized in a systematic, consistent or evidence-based manner across Kurdistan Region. Health care providers are often unaware of the existence of the BHSP. Primary care models and their respective content are not standardized or equitably distributed [30, 31, 53]. Delivery services, for example, are not provided in PHCCs [25]. Significant disparities have been documented to exist, particularly in relation to rural areas [77].

#### Standardizing the content of primary care

Three major health system performance assessment-oriented programs impacting the development of primary care in KRI have been the I-PSM program, USAID’s PHCPI, and the World Bank’s structural adjustment program. Each of these programs focus on standardizing the content and distribution of primary care, albeit leveraging differing strategies focusing on particular goals associated with UHC, MDGs, and structural economic reform [2, 7, 8].

A particularly interesting smaller-scale pilot project involved the recent implementation of a Dutch family practice model in Duhok [79]. Three Dutch family medicine doctors and two local family medicine doctor trainees provided family-oriented patient consultations using International Classification of Primary Care (ICPC)-based EMR systems. Physicians were assisted by specialized family medicine nurses and laboratory staff, who worked together to incorporate biopsychosocial variables into the primary care process.

### Primary care performance

#### Summary points

- *➢ Access: the economic crisis has negatively affected access to public primary care. Short opening hours, coupled with increased reliance on the public sector and irrational utilization have been associated with severe overcrowding.*
- *➢ Attachment and continuity: there is no general system of rostering to ensure first contact (continuity of) care with a regular primary care provider or team.*
- *➢ Comprehensiveness: the content of primary care delivery in Kurdistan Region is not routinely or systematically measured or evaluated.*
- *➢ Coordination: public primary care centers are not systematically networked, and standardized referral systems to secondary care are not well developed*
- *➢ Patient experiences: KRI’s population has a general preference for consulting specialists rather than GPs, due to perceptions of better quality of care and patient-provider interactions.*
- *➢ Provider experiences: physicians have been reported to generally express discontent in primary care settings. Nursing has been associated with professional dissatisfaction, intellectual isolation, perceived low social status and emigration.*

#### Access of primary care

Access is officially assessed by the travel time it takes for the average household within a respective population to reach a primary care center [80]. Although the majority of people live within 30 min of some type of primary care center, access is hindered by the fact that the public primary care system is operational only on a part-time basis (theoretically from 8 am to 2 pm) [19, 40, 53, 60]. The economic crisis has further reduced opening hours, from 7 days a week, to 5 days a week (operating reduced hours, between 8 am to 12 pm) [25].

Short opening hours, coupled with increased reliance on the public sector and irrational utilization have been associated with severe overcrowding [2]. Providers have associated overcrowding with inappropriate health care seeking behaviors due to poor health education and low consultation fees (~$0.20) [40, 60]. User fees have been reported to have recently been increased, in light of budgetary constraints [4].

Consultation times in public PHCCs have been documented to be inadequate, with reports that physicians tend to *“get rid of”* their public patients - who are often seen in groups, with little privacy - as quickly as possible, so as to devote themselves to their private practices [47].

#### Continuity of care

There is no general system of rostering to ensure continuity of care with a regular primary care provider or team [31, 53].

#### Comprehensiveness of primary care services

The content of primary care delivery in Kurdistan Region is not routinely or systematically measured or evaluated [53]. Furthermore, diagnostic and treatment procedures are generally not standardized, or distributed in accordance to population health needs. Limited resources and competencies limit the capability of health care providers to deliver basic health services (eg. less than a fifth of PHCCs in Sulaimaniya are providing Immunization, antenatal care & child growth monitoring), let alone effective team-based care within the context of multi-morbid patient populations with complex psychological and social problems [25, 31]. Updated and relevant clinical guidelines and decision aids are often not available, or properly used. Irrational treatment has been reported as a major problem by providers, attributed by physicians to time and resource constraints, along with a lack of job motivation [40, 60].

There also exist concerns relating to whether Iraq’s primary care system addresses the full range of women’s health care needs, particularly in rural areas. While primary care does focus on primarily on prenatal and maternal health, inadequate attention is given to adolescent girls and women’s reproductive health issues and concerns, including gender-based violence [81]. Furthermore, delivery services are not being offered by PHCCs, as required by the BHSP [33].

#### Coordination of care

Public primary care centers are not systematically networked, and standardized referral systems to secondary care are not well developed. Referrals usually consist of *“small, non-standardized handwritten notes”* [24, 53]. Providers have reported that patients often attend PHCCs, only to demand referral to particular hospitals (due to positive public perceptions of specialized care), which has been associated with over-referral [82].

Younis et al. (2014) reported that referral rates from PHCCs to hospitals in Duhok to be as high as 15.4% [83]. Furthermore, the referral note often fails to provide vital information (the name of the referring doctor was absent/unclear on 72.4% of a sample of 900 referral notes; only 22.4% of the referral notes provided a reason for the referral) [83]. The specialist doctors considered 52.8% of all referrals as inappropriate. The doctors from the PHCCs stated that 33% of their referrals were based on patient request, without sound medical reasons. This is similar to findings from two other studies in Erbil, which individually reported that 30.6% and 38.4% of all referrals were self-requested [84, 85].

#### Health outcomes and patient experiences

Outcomes of care and patient experiences (particularly in relation to primary care) are not routinely or systematically measured or evaluated, but have been anecdotally reported to be poor. A recent Q-methodology evaluation of patterns of health seeking behaviors and viewpoints relating to KRI’s public primary care services highlighted the general preference for consulting specialists rather than GPs, due to perceptions of better quality of care and patient-provider interactions.. Poor access to primary care services and organization of service provision were also often cited, including a lack of convenient waiting amenities, and poorly maintained hygienic environments [82]. Problems specific to the patient-provider interaction included lack of listening to patient problems, a lack of opportunity for patients to ask questions or obtain explanations for health problems andphysicians insufficiently explaining diseases and treatments. It is unclear whether these relational perceptions were limited to primary care, as they may also apply to specialist providers [82].

#### Primary care physician experiences

Apart from anecdotal reports, very little is known about the profiles and behaviors of physicians, and their interactions with patients in KRI. An ethnographic study of physician-patient interactions reported that “*common patterns of practice among physicians in Kurdistan include displays of discontent, reluctance to negotiate decisions with patients and unfavourable behaviours including dual practice and predatory behaviours towards patients. These behaviours are justified as a mechanism of dealing with negative aspects of their work, including overcrowding, low salaries and social pressure to live up to socially conceived ideas of a physician’s identity”* [47]. Physicians reported that rapid turnover is particularly problematic, due to factors such as poor retainment incentives, resource shortages, and a lack of training and career development opportunities [40, 41]. Negative reports about physician experiences and behaviors are tempered by findings in studies that indicate the hard-working character of many physicians, and their willingness to contribute to primary care development projects [30, 41].

#### Primary care nursing experiences

Within clinical settings, nurses generally have no job descriptions, are underutilized, and are usually limited to simple functions (ie. immunization), rather than health education, counseling, or other core medical services [31, 53, 86, 87]. The profession has been associated with professional dissatisfaction, intellectual isolation, perceived low social status and emigration [87]. These issues are particularly problematic in light of the fact that the majority of PHCCs, particularly those in rural and remote areas, are entirely staffed by nurses [53]. However, there has been increasing attention and productive efforts by nurses in KRI to develop their profession through training, research and advocacy [63, 64, 87].

## Discussion

The descriptive analysis of KRI’s primary care system brings to light key contextual variables, system strengths, weaknesses and developmental responses that are either common to transitioning middle-income countries, or idiosyncratic to the jurisdiction, affirming recent reviews of the literature [88]. The complex narrative comprises a rudimentary primary care system undergoing development, which is simultaneously under tremendous pressure to adapt to the continuously changing, complex and resource-intensive needs of sub-populations exhibiting varying morbidity patterns, within the context of protracted humanitarian, economic, political and social crises [4].

### System strengths

Strengths of KRI’s primary care system are generally overlooked, and have been the subject of only a few scientific investigations. Despite overall weak governance, there exists a KRG-governed public primary care system that has thus far demonstrated ongoing resilience in the face of multiple protracted crises. In the decade following the 2003 US invasion of Iraq, KRI increased the overall numbers of public primary care centers three times faster than the rest of Iraq, and had roughly four times more primary care centers than the rest of Iraq in 2012 (as a proportion of the population). From a governance perspective, the Directorates of Health have been perceived to be supportive of developing primary care in their local jurisdictions [41].

Structurally, there exists a network of urban and rural public primary care centers that are generally operational (albeit, sub-optimally resourced, managed and distributed), and sometimes able to deliver vertical programmatic services related to immunization, antenatal care, Integrated Management of Childhood Illness (IMCI) and diarrhea [31, 41]. Services offered are accessible to most of the population, from both a cost and geographical perspective.

Local health care providers have been actively participating in primary care strengthening initiatives such as the WHO’s IDHS-FPA program. KRI’s academic medical centers, in collaboration with international partners, have been continuously improving their primary care educational, research and patient care activities [60, 62]. KRI has also benefitted from the influx of highly trained Iraqi physicians, and the contribution of diaspora Kurdish professionals.

### System weaknesses and challenges

Weaknesses in the structures and processes of KRI’s primary care system are relatively characteristic of transitioning middle-income jurisdictions [88]. Notable results from this descriptive synthesis include: the structure and governance of dated Semashko models (ie. centralized, bureaucratic, non-data driven governance); a poorly regulated private sector, with high levels of private out-of- pocket expenditure; single-source, high risk public financing mechanisms (ie. oil revenue); weak funding mechanisms (ie. not accounting for activity, performance, care setting or patient complexity); a lack of timely, reliable and disaggregated data (ie. relating to health care needs and utilization); poorly developed human resources (ie. an emphasis on specialization; poor training programs; weak nursing profiles); poor governance and regulation of pharmaceuticals and health technologies; poor and unstandardized content, quality and distribution of primary care models (ie. lack of operationalization of an appropriate BHSP); a lack of rostering, medical record keeping and referral mechanisms; and poorly performing primary care delivery.

These findings are aligned to the results of qualitative studies in KRI, along with the outcomes of a recent meeting of Health Systems Global’s *‘Thematic Working Group on Fragile and Conflict-Affected States’* [4, 40, 41, 82] which focused on addressing health system challenges facing KRI within the context of ongoing crises, particularly in relation to primary care [4].

In addition to these weaknesses, the primary care system has to face the challenges of multiple protracted crises, notably the war on ISIS, the continued influx of IDPs and refugees, and the economic crisis. Thousands of Peshmerga and civilians have been killed or injured since the war with ISIS began in 2014. IDPs and refugees, the majority of whom reside with host communities in KRI, present extremely challenging epidemiological profiles (eg. infectious diseases, chronic illnesses, gender-based violence, PTSD, maternal and child health problems, physical injury, food, shelter and income insecurity). The economic crisis has not only decreased per capita public health care expenditure, but has simultaneously led to increasing utilization of the public sector by the population due to wage cuts and increasing unemployment. This is exacerbated by the fact that PHCCs are operating at reduced hours, staff capacity and with increasingly scarce resources.

### Critical appraisal of proposed responses

Many of the development-oriented responses proposed by both local (eg. the aforementioned working group) and international actors in KRI are those recently and commonly implemented in transitioning middle-income jurisdictions [4, 88, 89]. These include the development of alternative financing systems (eg. tax, social and private insurance systems), activity-based funding reforms, professionalization, development of health information systems, standardization of the content, organization, management and distribution of primary care services, and developing quality of care (eg. through performance assessment and evaluation).

However, the accompanying literature is often highly decontextualized, or specific to particular case studies, thereby providing little meaningful guidance in relation to the assumptions and critical factors underpinning successful operationalization [88, 90]. Furthermore, along with a paucity of studies relating to long-term cross-cutting outcomes (particularly from an equity lens), there exists the risk that policy-related performance metrics can be misappropriated and distorted by special interests, as often highlighted by Navarro. Nuanced societal consequences of policies (particularly from an equity perspective) can be purposely overlooked, or categorized as “unintended”.

These factors are particularly salient in relation to KRI, which manifests idiosyncratic contextual variables, weaknesses and responses that poorly fit the transitioning middle-income country development narrative. Politically, KRI is only semi-autonomous from Iraq, with a popular will for independence [1]. Despite the presence of democratic institutions, governance remains highly autocratic, with levels of corruption (particularly clientelism and nepotism) characterized by much lower-income countries [3]. Despite being middle-income from a pecuniary perspective, the oil and cash-based economy remains undiversified, with an extremely weak private sector, a bloated and ineffective public sector, and a dysfunctional banking system [20]. Furthermore, KRI’s economic dependency on Turkey and Iran has de facto rendered it, structurally, a low-income client state [10].

The security and humanitarian crises further set KRI apart from the middle-income developmental narrative. The confluence of crises drains vital developmental resources (ie. financial, human, social capital), while increasing the need for resource-intensive primary care for sub-populations exhibiting increasing demographic, socio-ethnic and epidemiological complexity. Humanitarian activities by authorities, aid and developmental organizations are increasingly being framed within developmental frameworks, with a focus on institutional development and building resilience within refugee, IDP and host communities and settings [38, 44]. Furthermore, development-oriented humanitarian approaches increasingly incorporate market-led development praxis, aligned with neoliberally-oriented approaches by actors such as the World Bank [36].

It is unclear and uncertain as to how the UHC-oriented (ie. WHO IDHS-FPA) and MDG-oriented (ie. USAID PHCPI) development programs will interact with the cross-cutting World Bank structural adjustment program, particularly within the context of the humanitarian emergency (ie. HRP and 3RP) programs. There is a need for scientific analyses of the synergies and tensions between the aforementioned approaches, and their specific impact and implications on the primary care system, along with the broader socioeconomic development and health status of Kurdistan Region.

The emphasis on neoliberal approaches requires further investigation, in light of the body of knowledge indicating that its doctrine is antithetical to the philosophy, theory and practice of primary care. Such approaches may run contrary to the primary care developmental processes premised on empowerment and self-determination [91].

What is clear, however, is the need for a common vision of primary care to underpin and align actions, and to enable synergy between all local and international actors involved in the development and humanitarian response.

### Study limitations

It is important to note that the descriptive analysis of KRI’s primary care system was limited by a paucity of relevant (scientific) literature, a lack of timely and validated statistics, and a reliance on the content of organizational publications that may contain biases or data of poor quality and reliability. Furthermore, due to the primary author’s limited Kurdish (Sorani), almost all data was compiled from English or Arabic language sources. The primary author is a PhD student is Canadian of Kurdish ethnicity, with an academic and professional background in health services research and social medicine. Potential biases in the selection and interpretation of data were adjudicated by the co-authors, who are also his PhD supervisors.

Findings were further triangulated against various data sources (when available), and completed and validated with different content knowledge experts, from different professional and organizational backgrounds, with a working knowledge of Kurdish and KRI’s health care system. Indeed, these experts provided useful local publications that the authors were previously unaware of.

## Conclusions

The resilience and sustainability of KRI’s primary care system is being threatened by the severe and protracted security, humanitarian, economic and political crises. Further complicating the situation is the fact that KRI is simultaneously experiencing largely uncoordinated primary care initiatives and reforms, being operationalized by a multitude of actors. The World Bank and RAND Corporation are implementing a cross-cutting World Bank structural economic adjustment program, typical of transitioning middle-income jurisdictions. The WHO continues to lead initiatives associated with the implementation of a Basic Health Services Package within family practice models, towards attainment of UHC. USAID has led work targeting MDGs 4 and 5. The Humanitarian Response Plan (HRP) and Regional Refugee and Resilience Plan (3RP), respectively led by the WHO and UNHCR, are operationalizing the emergency health care response for IDPs and refugees within developmental frameworks. A common vision of primary care is required to align resources, initiatives and policies, and to enable synergy between all local and international actors involved in the developmental and humanitarian response. The knowledge synthesized in this article can enable actors involved in KRI to align efforts, towards the ongoing development of primary care.

## Abbreviations

3RP: Regional, Refugee and Resilience Plan
BHSP: Basic Health Services Package
CME: Continuous Medical Education
DoH: Directorate of Health
GHDx: Global Health Data Exchange
HCDP: Human Capacity Development Program
HMIS: Health Management Information System
HRP: Humanitarian Response Plan
ICPC: International Classification of Primary Care
IDHS-FPA: Integrated District Health System Based on a Family Practice Approach
IDPs: Internally Displaced Persons
I-PSM: Iraq Public Sector Modernization Program
KMCA: Kurdistan Medical Control Agency
KRG: Kurdistan Regional Government
KRI: Kurdistan Region of Iraq
MDGs: Millennium Development Goals
MICS: Multiple Indicator Cluster Surveys
MoH: Ministry of Health
NHA: National Health Accounts
PHCC: Primary Health Care Center
PHCPI: USAID Primary Health Care Project in Iraq
UHC: Universal Health Coverage
UNICEF: UN Refugee Agency
WHO: World Health Organization
